# Neck Lymph Node Metastasis as A Poor Prognostic Factor in Thoracic Esophageal Squamous Cell Carcinoma Patients Receiving Concurrent Chemoradiotherapy: A Propensity Score-Matched Analysis

**DOI:** 10.1038/s41598-018-33400-3

**Published:** 2018-10-10

**Authors:** Yen-Hao Chen, Hung-I Lu, Chien-Ming Lo, Yu-Ming Wang, Shang-Yu Chou, Chang-Chun Hsiao, Li-Hsueh Shih, Su-Wei Chen, Shau-Hsuan Li

**Affiliations:** 1 Department of Hematology-Oncology, Kaohsiung Chang Gung Memorial Hospital and Chang Gung University College of Medicine, Kaohsiung, Taiwan; 2 Graduate Institute of Clinical Medical Sciences, College of Medicine, Chang Gung University, Taoyuan, Taiwan; 3 School of Medicine, Chung Shan Medical University, Taichung, Taiwan; 4 Department of Thoracic & Cardiovascular Surgery, Kaohsiung Chang Gung Memorial Hospital and Chang Gung University College of Medicine, Kaohsiung, Taiwan; 5 Department of Radiation Oncology, Kaohsiung Chang Gung Memorial Hospital and Chang Gung University College of Medicine, Kaohsiung, Taiwan; 6 Center for Shockwave Medicine and Tissue Engineering, Kaohsiung Chang Gung Memorial Hospital, Kaohsiung, Taiwan; 7 Department of Nursing, Kaohsiung Chang Gung Memorial Hospital, Kaohsiung, Taiwan; 8 Department of Anesthesia, National Cheng Kung University Hospital, Tainan, Taiwan

## Abstract

The present study investigated the clinical impact of neck lymph node (LN) metastasis in locally advanced inoperable thoracic esophageal squamous cell carcinoma (ESCC) patients who underwent concurrent chemoradiotherapy (CCRT) with a curative intent. There were 404 ESCC patients enrolled, including 35 patients with neck LN metastasis and 369 patients without such metastasis. Through the propensity score matching method, 35 patients of the 369 patients without neck LN metastasis were matched to the 35 patients with neck LN metastasis. Progression-free survival (PFS) and overall survival (OS) were found to be significantly worse in the neck LN metastasis group compared to the full non-neck LN metastasis group (9.8 months versus 5.9 months, P < 0.001, and 18.2 months versus 9.7 months, P = 0.001) and the matched non-neck LN metastasis group (9.9 months versus 5.9 months, P = 0.006, and 19.4 months versus 9.7 months, P = 0.007). In order to determine the difference between neck LN and supraclavicular LN metastasis, seventy patients with supraclavicular LN metastasis were also selected from the 369 patients without neck LN metastasis for comparison. Subsequently, when compared to the ESCC patients with supraclavicular LN metastasis, significantly worse PFS (8.5 months versus 5.9 months, P = 0.026) and OS (17.2 months versus 9.7 months, P = 0.047) were still found in the ESCC patients with neck LN metastasis. Our study indicates that neck LN metastasis is an independent poor prognostic factor for locally advanced inoperable thoracic ESCC patients who have undergone CCRT.

## Introduction

Esophageal cancer is known to be among the most aggressive human malignancies and is the ninth leading cause of cancer-related deaths in Taiwan, and esophageal squamous cell carcinoma (ESCC) accounts for up to 90% of all esophageal cancer cases^1^. The well-known risk factors of ESCC include smoking, alcohol consumption, long-term use of hot food and beverages, chewing betel quid, chronic mucosal irritation, achalasia, esophageal web, and a history of head and neck cancer^2–4^. More than half of ESCC patients have a locally advanced status with lymph node (LN) metastasis when the malignancy is diagnosed, resulting in a clinically unresectable disease and poor prognosis. At present, concurrent chemoradiotherapy (CCRT) is considered one of the standard therapeutic modalities for those locally advanced inoperable thoracic ESCC patients. Despite chemotherapy and radiotherapy having been remarkably improved, with significant advances for these ESCC patients in recent decades, the prognosis is still poor^5–9^.

LN metastasis is most common in ESCC patients, especially in those with locally advanced or metastatic status, and its bi-directional spread may extend from the neck to the celiac or retroperitoneal area. According to the definition of the 7^th^ edition of the American Joint Committee on Cancer (AJCC) staging system, neck LNs are regarded as non-regional or distant LNs in esophageal cancer, meaning that neck LN metastasis should be viewed as distant metastasis rather than regional LN metastasis^10^. On the other hand, although neck LNs are regarded as distant metastasis, the area of neck LN involvement is usually covered in the field of radiotherapy for those locally advanced ESCC patients receiving CCRT with curative intent. Furthermore, the neck area can typically tolerate higher doses of radiotherapy than the thoracic esophagus, resulting in better treatment response and an increased possibility of cure. Nevertheless, ESCC patients with neck LN metastasis have been classified as having stage IV disease and consequently have been excluded from most phase II or III clinical trials; therefore, the prognostic importance of neck LN metastasis in locally advanced inoperable thoracic ESCC patients still remains unclear^11,12^. Accordingly, there is no uniform opinion or standard care in the treatment of these ESCC patients with neck LN metastasis at present. As far as we know, the present study is the first to investigate the clinical significance of neck LN metastasis in such ESCC patients who have undergone CCRT with a curative intent.

In the current study, the locally advanced inoperable thoracic ESCC patients who received curative CCRT in our hospital were retrospectively reviewed, including those with neck LN metastasis but without distant metastasis. The aim of the present study was to evaluate the clinical impact of neck LN metastasis in locally advanced inoperable thoracic ESCC patients who have undergone CCRT with a curative intent.

## Results

### Clinicopathological characteristics

A total of 404 locally advanced inoperable thoracic ESCC patients who underwent CCRT with a curative intent were retrospectively examined from our ESCC database, including 35 ESCC patients with neck LN metastasis. The sample included 393 male patients and 11 female patients with a median age of 54 years (range, 35–85 years). Fourteen patients (3%) were diagnosed as having T1 status, 10 patients (2%) as having T2 status, 154 patients (39%) as having T3 status, and 226 patients (56%) as having T4 status. Meanwhile, 13 patients (3%) had N0 status, 178 patients (44%) had N1 status, 116 patients (29%) had N2 status, and 97 patients (24%) had N3 status. The primary tumor locations indicated that 143 patients (35%) had upper third ESCC, 163 patients (41%) had middle third ESCC, and 98 patients (24%) had lower third ESCC. Tumor grade analyses revealed 46 patients (12%) had grade 1 tumors, 179 patients (44%) had grade 2 tumors, and 179 patients (44%) had grade 3 tumors. The clinicopathological characteristics of these ESCC patients are shown in Table 1.

**Table 1:** Characteristics of 404 locally advanced inoperable thoracic esophageal squamous cell carcinoma patients receiving curative concurrent chemoradiotherapy.

Characteristics	
Age	54 years old (35–85)
Sex	
Male	393 (97%)
Female	11 (3%)
T status	
1	14 (3%)
2	10 (2%)
3	154 (39%)
4	226 (56%)
N status	
0	13 (3%)
1	178 (44%)
2	116 (29%)
3	97 (24%)
Grade	
1	46 (12%)
2	179 (44%)
3	179 (44%)
Location	
Upper	143 (35%)
Middle	163 (41%)
Lower	98 (24%)

### Clinical outcomes of neck LN metastasis

With respect to progression-free survival (PFS), a univariate analysis showed that age, tumor location, and tumor grade were not statistically significant predictors of PFS. Significantly better PFS (11.0 months versus 8.4 months, P = 0.019) was found in the 178 patients who had T1-3 status than in the 226 patients who had T4 status, and superior PFS (11.9 months versus 7.9 months, P = 0.001) was also found in the 192 patients with N0-1 status compared to the other 212 patients with N2-3 status. The 369 patients without neck LN metastasis were found to have superior PFS (9.8 months versus 5.9 months, P < 0.001) in comparison with the 35 patients with neck LN metastasis. In a multivariate analysis, female sex (P = 0.046, hazard ratio (HR): 0.50, 95% confidence interval (CI): 0.26–0.99), T1-3 status (P = 0.019, HR: 0.78, 95% CI: 0.64–0.96), N0-1 status (P = 0.022, HR: 0.78, 95% CI: 0.63–0.97), and non-neck LN metastasis status (P = 0.006, HR: 0.59, 95% CI: 0.41–0.86) were the independent prognostic parameters of better PFS.

With respect to overall survival (OS), a univariate analysis indicated that sex, age, tumor location, and tumor grade were not statistically significant predictors of OS. Significantly better OS (21.2 months versus 14.8 months, P = 0.030) was found in the 178 patients who had T1-3 status than in the 226 patients who had T4 status, and superior OS (23.3 months versus 13.6 months, P = 0.020) was also found in the 192 patients with N0-1 status in comparison to the 212 patients with N2-3 status. The 369 patients without neck LN metastasis were found to have better OS (18.2 months versus 9.7 months, P = 0.001) compared to the 35 patients with neck LN metastasis. A multivariate analysis showed that T1-3 status (P = 0.028, HR: 0.78, 95% CI: 0.63–0.97) and non-neck LN metastasis status (P = 0.001, HR: 0.53, 95% CI: 0.36–0.78) were the independent prognostic parameters of better OS. These univariate and multivariate survival analyses are shown in Table 2.

**Table 2:** Univariate and multivariate analysis of progression-free survival and overall survival in in 404 locally advanced inoperable thoracic esophageal squamous cell carcinoma patients receiving curative CCRT.

Characteristics	No. of patients	Univariate analysis		Multivariate analysis		Univariate analysis		Multivariate analysis	
		Median PFS (months)	P value	HR (95% CI)	P value	Median OS (months)	P value	HR (95% CI)	P value
Age									
<60 years	296 (73%)	9.2	0.23			15.7	0.57		
≥60 years	108 (27%)	8.8				18.4			
Sex									
Male	393 (97%)	9.1	0.015*			16.2	0.16		
Female	11 (3%)	17.3		0.50 (0.26–0.99)	0.046*	40.0			
T status									
1 + 2 + 3	178 (44%)	11.0	0.019*	0.78 (0.64–0.96)	0.019*	21.2	0.030*	0.78 (0.63–0.97)	0.028*
4	226 (56%)	8.4				14.8			
N status									
0 + 1	192 (47%)	11.9	0.001*	0.78 (0.63–0.97)	0.022*	23.3	0.020*		
2 + 3	212 (53%)	7.9				13.6			
Grade									
1 + 2	225 (56%)	8.4	0.36			15.2	0.54		
3	179 (44%)	10.4				19.2			
Location									
Upper	143 (35%)	8.4	0.59			14.8	0.30		
Middle + Lower	261 (65%)	9.9				17.6			
Neck LN metastasis									
Yes	35 (9%)	5.9	<0.001*			9.7	0.001*		
No	369 (91%)	9.8		0.59 (0.41–0.86)	0.006*	18.2		0.53 (0.36–0.78)	0.001*

CCRT: concurrent chemoradiotherapy; LN: lymph node; PFS: progression-free survival; OS: overall survival; HR: hazard ratio; CI: confidence interval. *Statistically significant.

### Comparison of ESCC patients with/without neck LN metastasis

According to neck LN metastasis status, the 404 locally advanced inoperable thoracic ESCC patients were divided into two groups: the 35 patients with neck LN metastasis (the neck LN metastasis group) and the other 369 patients without such metastasis (the non-neck LN metastasis group). There were no significant differences in the baseline characteristics of these two groups, except for tumor T status (P = 0.001), tumor N status (P < 0.001), tumor grade (P < 0.001), and tumor location (P = 0.004). Furthermore, there was a higher percentage of cases with more advanced N status and upper third tumor location in the neck LN metastasis group than the non-neck LN metastasis group.

Using the propensity score matching method, 35 matched patients of the 369 ESCC patients without neck LN metastasis were identified to compare to the neck LN metastasis group. Parameters including tumor T status, tumor N status, age, sex, tumor location, and tumor grade were all matched so that no statistical difference was seen between these two groups (Table 3).

**Table 3:** Clinicopathological parameters in 404 locally advanced inoperable thoracic esophageal squamous cell carcinoma patients with/without neck LN metastasis receiving curative CCRT.

Characteristics	Neck LN metastasis group (N = 35)	Non-neck LN metastasis group (N = 369)	P value
Age			
<60 years	27 (77%)	269 (73%)	0.59
≥60 years	8 (23%)	100 (27%)	
Sex			
Male	35 (100%)	358 (97%)	0.30
Female	0 (0%)	11 (3%)	
T status			
1	5 (14%)	9 (2%)	0.001*
2	1 (3%)	9 (2%)	
3	7 (20%)	147 (41%)	
4	22 (63%)	204 (55%)	
N status			
0	0 (0%)	13 (3%)	<0.001*
1	5 (14%)	174 (47%)	
2	9 (26%)	106 (29%)	
3	21 (60%)	76 (21%)	
Grade			
1	4 (11%)	42 (11%)	<0.001*
2	29 (83%)	150 (41%)	
3	2 (6%)	177 (48%)	
Location			
Upper	21 (60%)	122 (33%)	0.004
Middle	11 (31%)	152(41%)	
Lower	3 (9%)	95 (26%)	
**Characteristics **	**Neck LN metastasis group (N = 35) **	**#Matched non-neck LN metastasis group (N = 35) **	**P value **
Age			
<60 years	27 (77%)	27 (77%)	1.0
≥60 years	8 (23%)	8 (23%)	
Sex			
Male	35 (100%)	35 (100%)	1.0
Female	0 (0%)	0 (0%)	
T status			
1	5 (14%)	5 (14%)	1.0
2	1 (3%)	1 (3%)	
3	7 (20%)	7 (20%)	
4	22 (63%)	22 (63%)	
N status			
0	0 (0%)	0 (0%)	1.0
1	5 (14%)	5 (14%)	
2	9 (26%)	9 (26%)	
3	21 (60%)	21 (60%)	
Grade			
1	4 (11%)	4 (11%)	1.0
2	29 (83%)	29 (83%)	
3	2 (6%)	2 (6%)	
Location			
Upper	21 (60%)	21 (60%)	1.0
Middle	11 (31%)	11 (31%)	
Lower	3 (9%)	3 (9%)	

LN: lymph node; CCRT: concurrent chemoradiotherapy; ^#^Using propensity score matching method. *Statistically significant.

**Figure 1:**
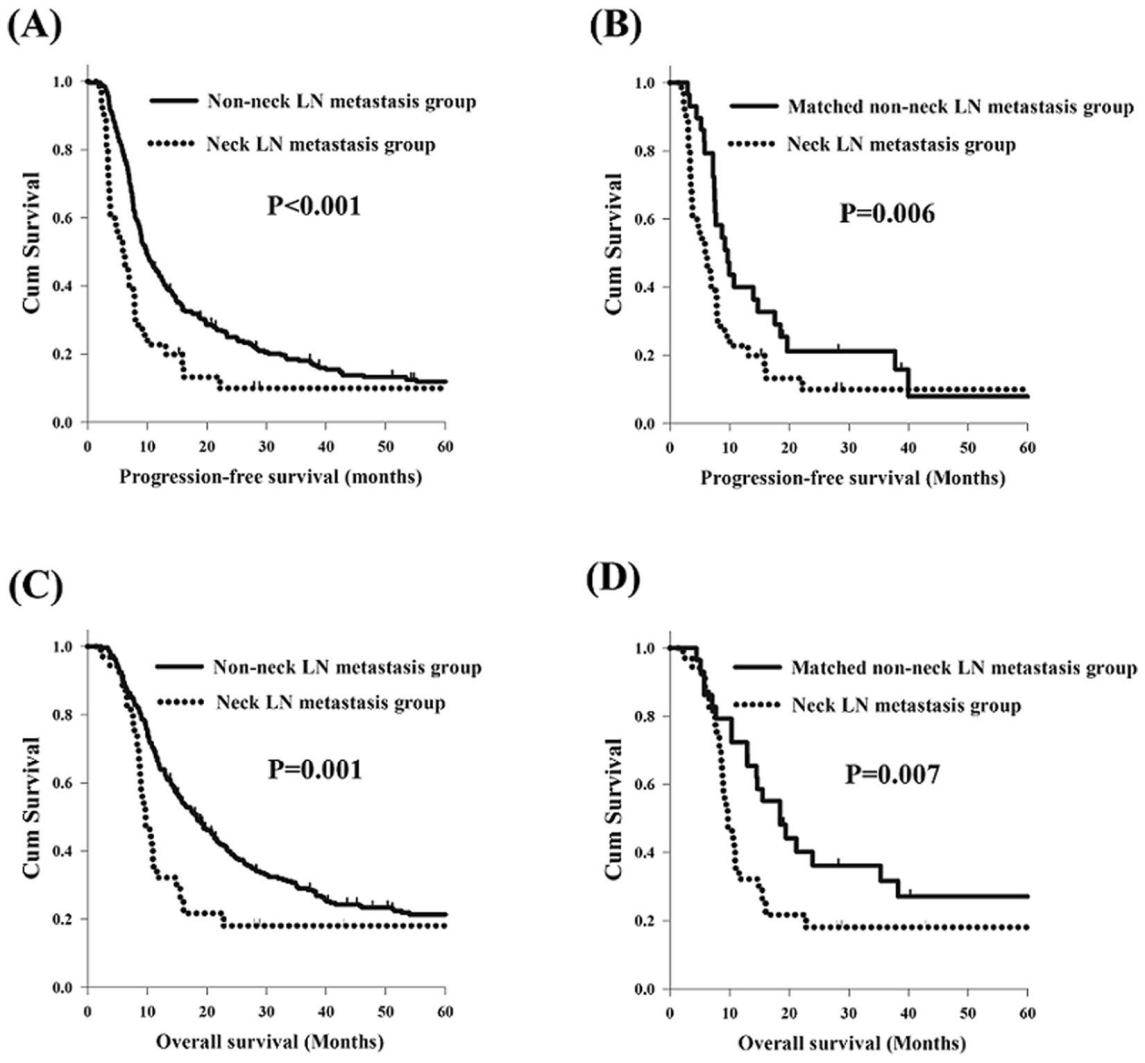
The survival curves of esophageal squamous cell carcinoma patients with/without neck lymph node (LN) metastasis. (**A**) Progression-free survival, neck LN metastasis group versus non-neck metastasis LN group. (**B**) Progression-free survival, neck LN metastasis group versus matched non-neck LN metastasis group (matched using the propensity score matching method). (**C**) Overall survival, neck LN metastasis group versus non-neck LN metastasis group. (**D**) Overall survival, neck LN metastasis group versus matched non-neck metastasis LN group (matched using the propensity score matching method).

Worse PFS was found in the neck LN metastasis group (N = 35) compared to the full non-neck LN metastasis group (N = 369, 5.9 months versus 9.8 months, P < 0.001, Fig. 1A) and the matched non-neck LN metastasis group (N = 35, 5.9 months versus 9.9 months, P = 0.006, Fig. 1B). Moreover, worse OS was also found in the neck LN metastasis group (N = 35) than the full non-neck LN metastasis group (N = 369, 9.7 months versus 18.2 months, P = 0.001, Fig. 1C) and the matched non-neck LN metastasis group (N = 35, 9.7 months versus 19.4 months, P = 0.007, Fig. 1D).

Among all 404 locally advanced inoperable thoracic ESCC patients, there were 383 patients who were reported to have experienced treatment failure, including 32 patients in the neck LN metastasis group and 351 patients in the non-neck LN metastasis group. There was a higher percentage of distant metastasis in the neck LN metastasis group in comparison with the non-neck LN metastasis group (53% versus 28%, P = 0.03).

### Comparison between ESCC patients with neck LN metastasis and supraclavicular LN metastasis

**Figure 2:**
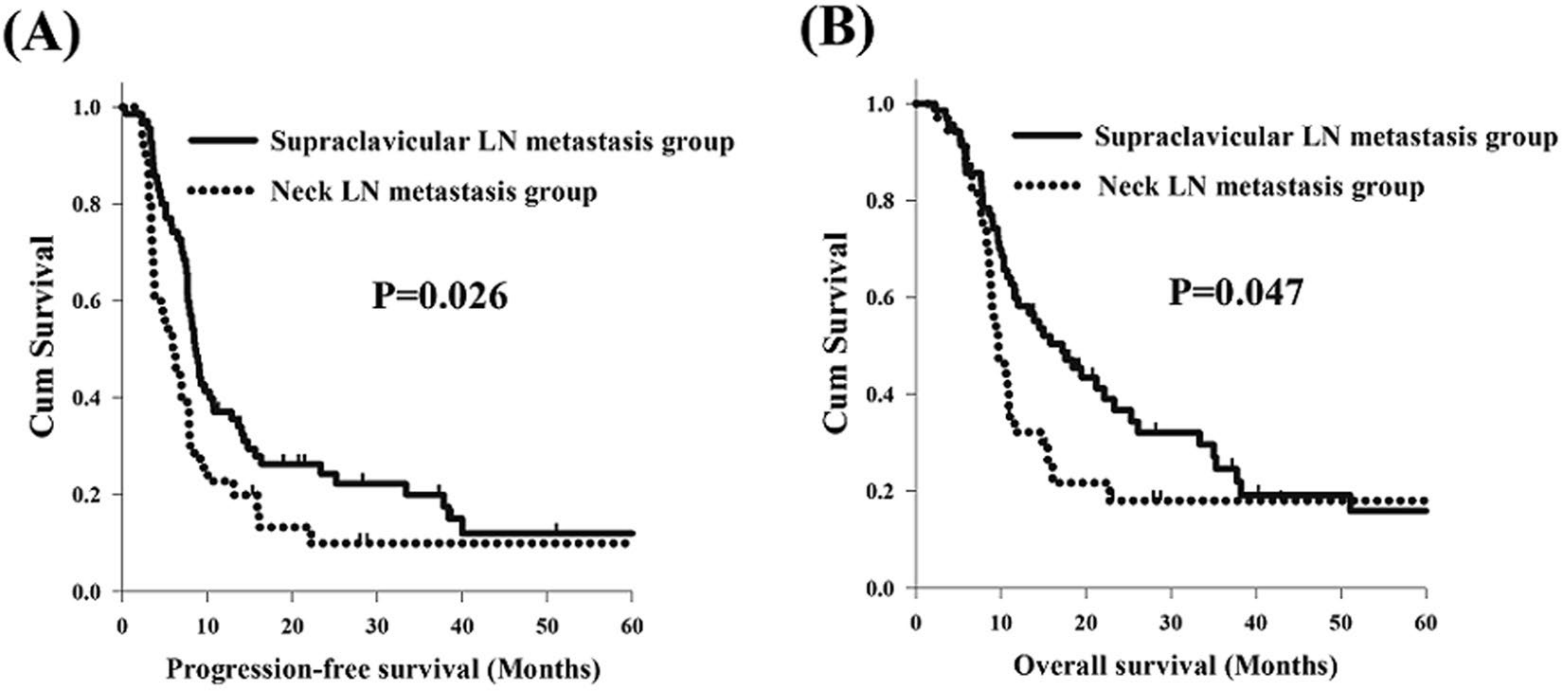
Survival comparison between esophageal squamous cell carcinoma patients with neck and supraclavicular lymph node metastasis. (**A**) progression-free survival. (**B**) overall survival.

Several previous studies have revealed that supraclavicular LN metastasis is not an independent prognostic factor for the survival of esophageal cancer patients^13–15^. However, the location of supraclavicular LNs is near the area of neck LNs in terms of anatomical distribution. Thus, we also examined whether any significant difference may exist in survival between the neck LN metastasis group and the supraclavicular LN metastasis group. There were 35 patients in the neck LN metastasis group and 70 patients in the supraclavicular LN metastasis group, and the baseline parameters were similar without any significant difference between these two groups (Table 4). Worse PFS (5.9 months versus 8.5 months, P = 0.026, Fig. 2A) and OS (9.7 months versus 17.2 months, P = 0.047, Fig. 2B) were found in the patients with neck LN metastasis in comparison to those with supraclavicular LN metastasis.

**Table 4:** Clinicopathological parameters in 105 locally advanced esophageal squamous cell carcinoma patients with neck or supraclavicular LN metastasis.

Characteristics	Neck LN metastasis group (N = 35)	Supraclavicular LN metastasis group (N = 70)	P value
Age			
<60 years	27 (77%)	53 (76%)	0.87
≥60 years	8 (23%)	17 (24%)	
Sex			
Male	35 (100%)	67 (96%)	0.21
Female	0 (0%)	3 (4%)	
T status			
1 + 2 + 3	13 (37%)	33 (47%)	0.33
4	22 (63%)	37 (53%)	
N status			
0 + 1	5 (14%)	12 (17%)	0.71
2 + 3	30 (86%)	58 (83%)	
Grade			
1 + 2	33 (94%)	64 (91%)	0.60
3	2 (6%)	6 (9%)	
Location			
Upper	14 (40%)	35 (50%)	0.33
Middle + Lower	21 (60%)	35 (50%)	

LN: lymph node.

## Discussion

ESCC patients with neck LN metastasis account for only a small percentage of ESCC patients overall. According to the definition of the 7^th^ AJCC staging system, neck LNs are regarded as non-regional and distant LNs in thoracic esophageal cancer and are viewed as stage IV disease^10^. Therefore, ESCC patients with neck LN metastasis were excluded in most previous clinical trials, resulting in the prognostic importance of neck LN metastasis having not been clearly determined^11,12,16^. In general, for ESCC patients with distant metastasis, palliative chemotherapy or the best supportive care possible is the preferred standard care in clinical practice. However, neck LN metastasis is different from distant visceral metastasis. First, the area of neck LNs is near supraclavicular LNs in terms of anatomical position, and this area is usually covered in the field of radiotherapy for those locally advanced ESCC patients receiving CCRT with curative intent. Second, the neck area can typically tolerate higher doses of radiotherapy than the thoracic esophagus, resulting in better treatment response and an increased possibility of cure. CCRT and esophagectomy with three-field LN dissection are usually the standard treatment modalities for ESCC patients with supraclavicular LN metastasis. Additionally, for synchronous ESCC and head/neck cancer patients, CCRT is frequently the one of the best treatment options if surgical resection is unfeasible^17^. Therefore, there is the potential of cure for these locally advanced inoperable thoracic ESCC patients with neck LN metastasis without distant visceral metastasis when curative CCRT is performed. So far as we know, however, for ESCC patients with neck LN metastasis, there has been no evidence provided that has focused on the capacity and prognosis of the different therapeutic modalities. Thus, the present study is the first to investigate the prognostic importance of neck LN metastasis in locally advanced inoperable thoracic ESCC patients who have undergone CCRT with a curative intent.

In this study, worse PFS and OS were found in the ESCC patients with neck LN metastasis in comparison to those without neck LN metastasis, whether in the univariate or multivariate Cox regression analysis. Moreover, in order to correct for bias, we used the propensity score matching method to select a control group from among the locally advanced ESCC patients without neck LN metastasis treated with curative CCRT in our hospital. The comparison of this matched group of non-neck LN metastasis patients with the neck LN metastasis patients yielded results similar to those of the comparison between the neck LN metastasis patients and all 369 non-neck LN metastasis patients, further indicating that neck LN metastasis was a strong prognostic parameter of poor outcome compared to non-neck LN metastasis. Furthermore, a higher percentage of patients with distant visceral metastasis was found in the neck LN metastasis group in comparison to the non-neck LN metastasis group.

The prognosis of ESCC patients is related to the area of LN metastasis and tumor location. According to the definition of the 6^th^ edition of the AJCC staging system, celiac LNs are regarded as distant and non-regional LNs in thoracic esophageal cancer. For lower third esophageal cancer, celiac LN metastasis was considered an M1a disease; for upper and middle third esophageal cancer, celiac LN metastasis was defined as an M1b disease^18^. Nevertheless, celiac LNs were re-defined as regional LNs and the M1a and M1b classifications were removed in the 7^th^ AJCC staging system^10^. A previous study showed that tumor location was related to PFS and OS in ESCC with celiac LN metastasis, indicating that better PFS and OS were found in lower third ESCC patients than in those with upper and middle third ESCC^19^. However, according to the definitions of both the 6^th^ and 7^th^ editions of the AJCC staging system, neck LNs are defined as non-regional LNs, even though they are usually included in the radiotherapy field and could be treated with higher radiotherapy doses. In this study, while there was a higher percentage of neck LN metastasis in upper third ESCC patients, no significant difference in PFS or OS was found among the groups with different tumor locations.

In recent years, growing evidence has shown that supraclavicular LN metastasis is not associated with poor prognosis^13,14,20,21^. Cho *et al*. revealed that supraclavicular LN metastasis was not a prognostic parameter for either PFS or OS in ESCC patients who underwent neoadjuvant chemoradiotherapy and esophagectomy^13^. A Dutch study demonstrated that the number of affected LNs is an important independent prognostic factor for esophageal cancer patients treated with definitive chemoradiation, whereas the involvement of a supraclavicular LN is not^14^. A Japanese study, reported by Tachimori *et al*., indicated that compared to the area of the involved nodes, the number of involved nodes is the most predictive parameter for postoperative survival in ESCC patients who have undergone esophagectomy and three-field LN dissection^21^. Miyata *et al*., meanwhile, reported that preoperative treatment, such as chemotherapy, can change the status of supraclavicular LN metastasis, resulting in the possibility of curative surgery^20^. Therefore, because the area of neck LNs is near supraclavicular LNs in terms of anatomical position and is usually covered in the field of radiotherapy for those locally advanced inoperable thoracic ESCC patients who have undergone curative CCRT, we wanted, in this study, to determine whether there is any difference in clinical outcomes between neck LN metastasis and supraclavicular LN metastasis in ESCC patients treated with CCRT with a curative intent. The present study showed that neck LN metastasis is more aggressive than supraclavicular LN metastasis, and that patients with neck LN metastasis had a worse prognosis, generally speaking, than those with supraclavicular LN metastasis, whether in terms of PFS or OS.

There are some limitations in our current study. First, the study was a retrospective review with a small sample size, and all patients were treated at a single institution. Second, there was a limited number of female patients, such that there is bias in the comparison of survival outcome between male and female patients. However, as far as we know, the current study is the first study to investigate the prognostic significance of neck LN metastasis. Furthermore, it comprises the largest series thus far of locally advanced inoperable thoracic ESCC patients with neck LN metastasis who received CCRT with a curative intent, and may be helpful to clarify the situation of this rare ESCC group.

In conclusion, our study suggests that neck LN metastasis is an independent prognostic factor for locally advanced inoperable thoracic ESCC patients who have undergone CCRT with a curative intent.

## Methods

### Patient population

A cohort of 1,045 ESCC patients with available medical records who underwent treatment between January 2000 and December 2015 at Kaohsiung Chang Gung Memorial Hospital were retrospectively reviewed. First, patients with distant metastasis other than neck LN metastasis, celiac LN metastasis, or a history of second primary malignancy, such as head and neck cancers, were excluded. Subsequently, only the patients who underwent CCRT with curative intent were included. That is, any patients who were treated with other therapeutic protocols, such as esophagectomy followed by adjuvant chemotherapy/radiotherapy, palliative radiotherapy or chemotherapy, or best supportive care, were excluded. Finally, there were 404 ESCC patients who met the criteria for further analysis, including 35 patients who had neck LN metastasis without distant metastasis. Thus, these 404 locally advanced ESCC patients were divided into two groups: a neck LN metastasis group consisting of the aforementioned 35 patients and a non-neck LN metastasis group consisting of the other 369 patients.

In order to prevent selection bias, the propensity score matching method was performed among the 369 ESCC patients without neck LN metastasis. First, a propensity score was calculated using binary logistic regression, with covariates including tumor T status, tumor N status, tumor location, tumor grade, age, and sex being entered in the propensity model. After that, a 1-to-1 match with the closest matching scores between the 35 patients with neck LN metastasis and 35 patients without neck LN metastasis was determined, such that the 35 matched ESCC patients without neck LN metastasis could then be regarded as a control group for the patients with neck LN metastasis.

**Figure 3:**
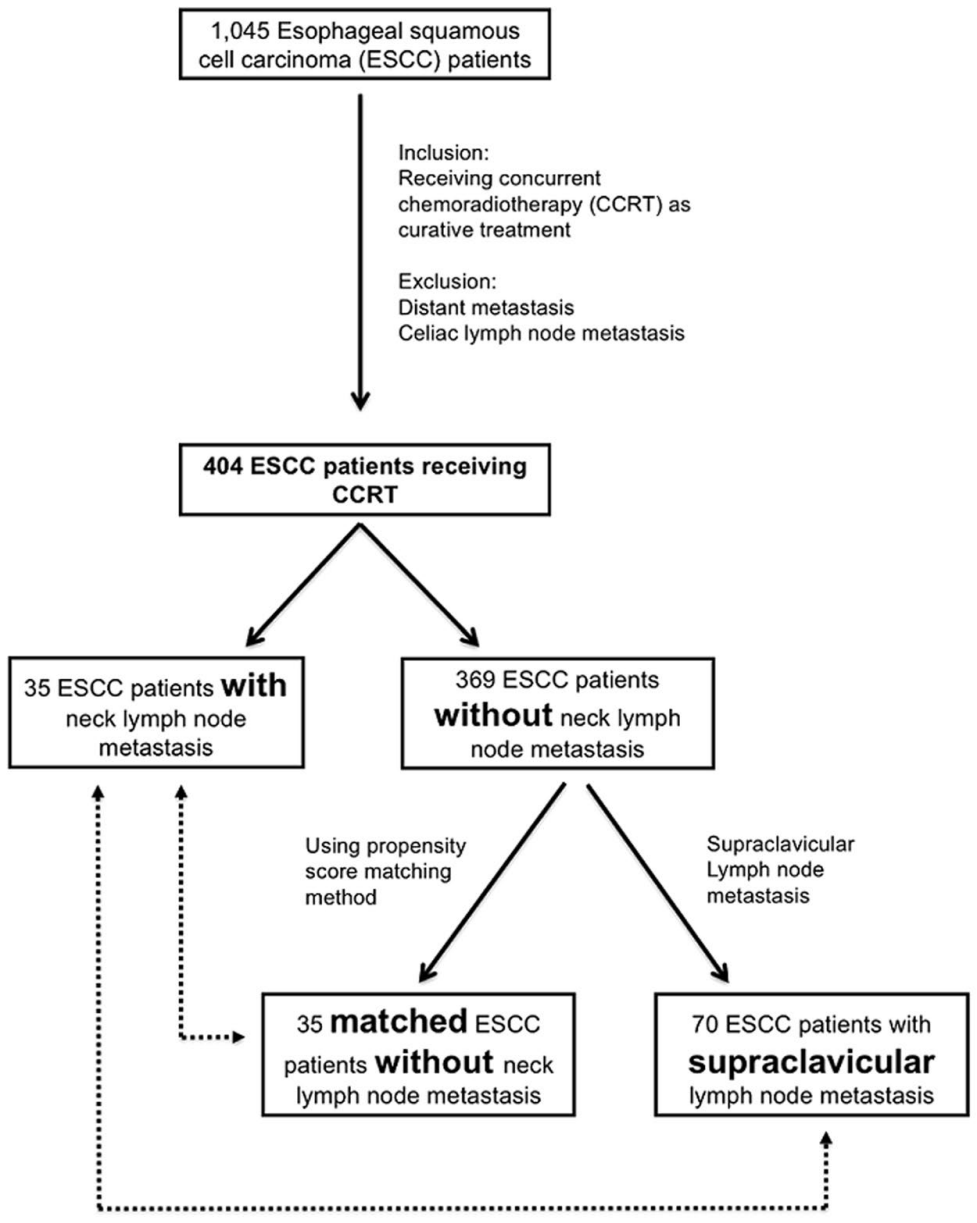
Algorithm for identifying locally advanced inoperable thoracic esophageal squamous cell carcinoma (ESCC) patients with or without neck lymph node metastasis.

Moreover, among the 369 ESCC patients without neck LN metastasis, 70 patients with supraclavicular LN metastasis were selected to be compared to those with neck LN metastasis in terms of clinical outcomes. The algorithm used is shown in Fig. 3.

### Definition of neck/supraclavicular lymph nodes and clinical tumor stage

Each ESCC patient in our study received endoscopic ultrasonography (EUS), chest computed tomography (CT), and positron emission tomography (PET) scans to determine the clinical tumor stage according to the 7^th^ AJCC staging system. The identifications of neck and supraclavicular LNs were based on the RTOG guideline^22^.

Neck LNs were defined as those LNs situated in the areas below: level I (submental and submandibular nodes), level II (upper jugular nodes), level III (middle jugular nodes), level IVa (lower jugular nodes), level V (nodes in the posterior triangle), and level VIa (anterior jugular nodes). LNs located in the level IVb (medial supraclavicular nodes) and level Vc (lateral supraclavicular nodes) were regarded as supraclavicular LNs. Metastasis-positive LNs had to meet one of the following criteria: first, be spherical and have a maximum transverse diameter > 10 mm with a short-axis diameter >5 mm on a chest CT scan^23^; or, second, have a high uptake of 18F-fluorodeoxyglucose detected by a PET scan with a maximum standardized uptake value > 4.1^24^.

### Chemotherapy and radiotherapy

The details of our curative intent radiotherapy are described below. For each patient, a customized thermoplastic immobilization device was designed. The patient then underwent CT-simulation for image acquisition. As the treatment field covered the neck and mediastinum, inverse plan intensity-modulated radiotherapy (IMRT) was used to deliver 6- or 10-MV photons. According to chest CT scan and/or PET-CT images, the definition of the gross target volume (GTV) was the gross tumor and gross LNs. The clinical target volume (CTV) comprehensively covered the esophagus, the mediastinal LNs, the bilateral neck, and the supraclavicular LNs. The planning target volume (PTV) was expended from the CTV with 0.5–1.0 cm margins in all directions. The total dose prescribed to the PTV was 50–50.4 Gy in 25–28 daily fractions followed by a boost dose to the gross neck LNs for an additional 10–16 Gy in 5–8 daily fractions.

Chemotherapy consisted of cisplatin (75 mg/m^2^; 4-hour infusion) on day 1 and 5-fluorouracil (1000 mg/m^2^; continuous infusion) on days 1–4 every 4 weeks, and was arranged concurrently with radiotherapy. For patients with creatinine clearance <60 mL/min, carboplatin was used instead of cisplatin.

### Statistical analysis

Statistical analyses were conducted with the SPSS 19 software package (IBM, Armonk, NY). Statistical analyses of group differences were performed using the *t*-test, Fisher’s exact test, and chi-square test for categorical variable data. PFS was defined as the time from starting treatment to disease progression or death from any cause; OS was calculated from the date of diagnosis of the esophageal cancer to death or last contact.

The Kaplan–Meier method was used to estimate PFS and OS, and the log rank test was performed to evaluate the differences between groups for univariate analysis. Multivariate analysis was described with HRs and their 95% CI values derived from the Cox proportional hazards model, which were used to quantify the associations between the prognostic factors and clinical outcome. All of the tests were two-sided tests, and P < 0.05 was considered statistically significant.

### Ethics statement

The study was approved by the Chang Gung Medical Foundation Institutional Review Board (201700721B0). All the methods were carried out in accordance with the approved guidelines, and written informed consent of the patients or their families was not judged necessary for this kind of retrospective study by the Chang Gung Medical Foundation Institutional Review Board.
